# Exploring the Interaction of RBD with Human β Defensin Type 2 Point Mutants: Insights from Molecular Dynamics Simulations

**DOI:** 10.1021/acs.jpcb.4c07004

**Published:** 2025-02-10

**Authors:** Ishrat Jahan, Liqun Zhang

**Affiliations:** Chemical Engineering Department, 4260 University of Rhode Island, Kingston, Rhode Island 02881, United States

## Abstract

The global health crisis triggered by the SARS-CoV-2 virus has highlighted the urgent need for effective treatments. As existing drugs are not specifically targeted at this virus, there is a growing interest in exploring natural antimicrobial peptides such as defensin as potential therapeutic options. Human β defensin type 2 (hBD-2), which is a cationic cysteine-rich peptide, serves as the initial barrier against bacterial and fungal invaders in mammals. It can bind with Spike-RBD and occupy the same site as the ACE2 receptor, thereby hindering viral entry into cells expressing ACE2. To explore the effect of different point mutations on the binding of hBD-2 with RBD, the binding dynamics and interactions between hBD-2 point mutants with RBD were studied and compared with that of RBD&hBD-2 wild-type complex. In total, 247 hBD-2 point mutants were built with the mutation sites at the binding region of hBD-2 (RES18–30) with the RBD of CoV-2. All-atom molecular dynamics simulations were carried out on RBD binding with hBD-2 point mutants. Analysis based on root-mean-square deviation (RMSD), hydrogen bonds analysis, and binding free energy using the MM/PBSA method revealed that many point mutants of hBD-2 exhibit weaker binding with RBD compared to the wild type; however, a subset of mutants, including C20I, C20K, R22W, R23H, R23L, Y24L, K25F, K25H, G28Y, T29R, and C30K, displayed enhanced binding with RBD. The findings can offer insights designing hBD-2-based novel drugs to combat SARS-CoV-2 in the long term.

## Graphical abstract

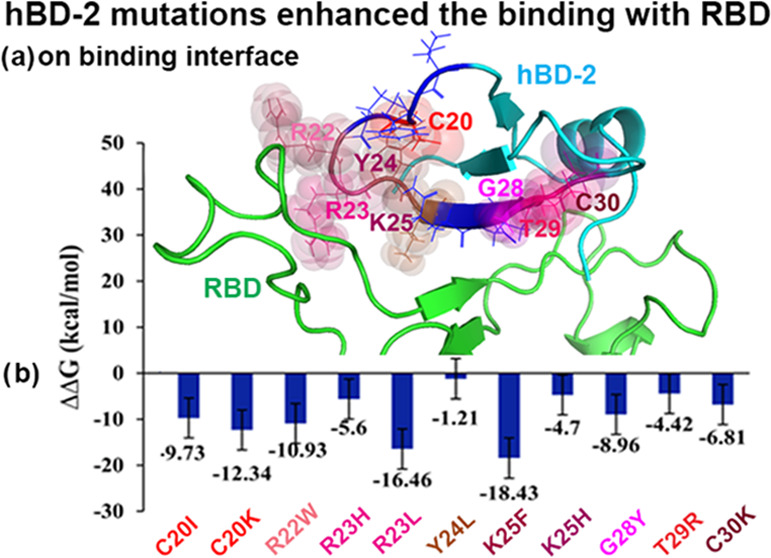

## Introduction

The appearance of Severe Acute Respiratory Syndrome Coronavirus 2 (SARS-CoV-2) has introduced an unprecedented global health dilemma, emphasizing the essential requirement for a thorough comprehension of viral–host interactions to develop effective treatments.^1^ Coronaviruses, recognized for their large size and enveloped structure, are RNA viruses with a positive strand known for their swift mutation rate, which influences how they spread. Despite the rollout of vaccines, these viruses persist in adapting, potentially developing increased transmissibility or enhanced resistance to immunity acquired from previous infection or vaccination.^2^ A crucial element of coronaviruses is the Spike (S) protein, which binds to host cell receptors and facilitates viral fusion with cell membranes.^3–5^ This binding process involves dynamic alterations within the receptor-binding domain (RBD) of the S protein,^6^ allowing interaction with the angiotensin-converting enzyme 2 (ACE2) receptor on host cells.^7–9^ With respect to anti-SARS-CoV-2 infection, defensins, which are key components of the human immune system,^10,11^ have been explored as potential therapeutic options.^12^

Natural antimicrobial peptides (AMPs) are gaining attention for their effectiveness against viruses.^13^ Human-derived AMPs like defensins and cathelicidin LL-37 play a key role in early defense against lung viruses.^14^ Human α defensin type 5 (HD5), LL-37, and human β defensin type 2 (hBD-2) have shown promise in blocking CoV-2 invasion, indicating potential as antiviral treatments.^15–17^ hBD-2 is a cationic antimicrobial peptide that acts as the body’s first defense against bacteria and fungi. It is primarily located in human mucosal surfaces, such as the respiratory tract, and is part of the innate immune system. hBD-2 serves as the initial barrier against various pathogens like bacteria, viruses, and fungi, disrupting their cell membranes and inhibiting their growth.^2,5^ During CoV-2 virus infection process, through binding to the ACE2 receptor, the RBD of the Spike protein is essential in promoting viral entrance into host cells.^7–9,18,19^ In the context of blocking viral infections, comprehending the plausible interplay between hBD-2 and RBD is important because it can provide valuable perspectives on the innate immune systems against viral infections, as well as possible treatment approaches. From ΔΔ*G* predictions in our former project,^2^ hBD-2 should have a similar neutralization effect on the α variant as on the original SARS-CoV-2 virus, and the β variant should be able to escape the neutralization effect of hBD-2. In order to design hBD-2-based novel drugs that can have a stronger binding affinity with RBD than the hBD-2 wild type, studying the binding and interaction between hBD-2 mutants and RBD is essential. In this study, we focus on exploring the impact of point mutations at the hBD-2 binding interface on its interactions with RBD of the Spike protein, by systematically mutating each residue in the region of RES18–30 on hBD-2 to all other 19 amino acids. We seek to uncover potential changes in the hBD-2&RBD interaction landscape. Our approach allows us to probe the structural and dynamic consequences of these mutations, providing valuable insights into the molecular determinants governing the hBD-2&RBD interaction.

## Methods

### Molecular Dynamics Simulations

The complex structure of hBD-2 ^20^ bound with RBD is taken from our previous work,^17^ as shown in Figure 1, which has the mutation sites on hBD-2 highlighted and residues labeled in red.

**Figure 1:**
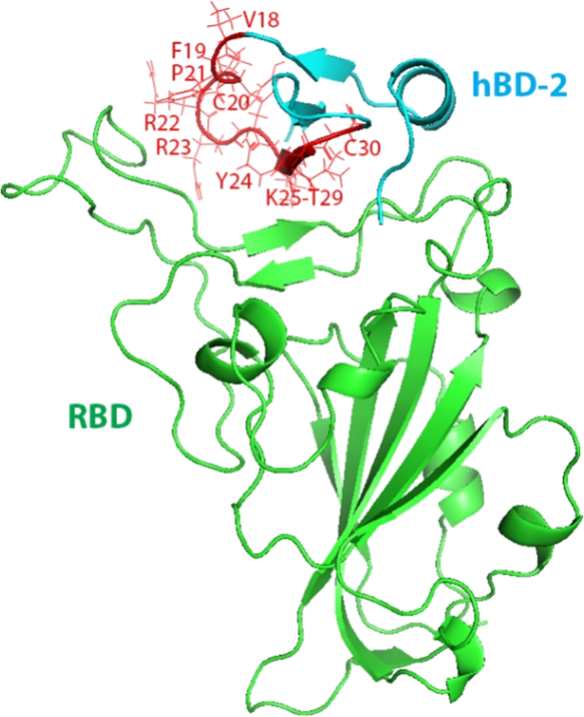
Binding structure of hBD-2 (cyan) with RBD (green) used as a starting structure for the MD simulations in wild type (WT) and for spot mutants with the binding region of V18–C30 highlighted in red lines and mostly labeled.

The hBD-2 sequence is:


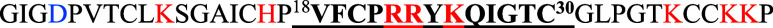


The 13 residues in bold and underlined (RES18–RES30) are at the binding interface with RBD^17^ as highlighted in red lines in Figure 1. The positively charged residues are highlighted in red, while negatively charged residues are in blue in the hBD-2 sequence. Each amino acid in the binding region of hBD-2 (RES18–30) is mutated to 19 other amino acids to prepare the hBD-2 point mutants using pymol software.^21^ Then, all-atom molecular dynamics (MD) simulations were carried out for the point mutant hBD-2& RBD (in total 247 simulations) besides the repeat of the hBD-2&RBD in wild type. Thus, a total of 248 MD simulations were performed using the GROMACS-2023.1 ^22^ package with CHARMM36-jul2022 force field.^23^ During the simulations, disulfide bonds between the cysteine residues (Cys11–Cys40, Cys18–Cys33, Cys23–Cys41) were conserved whenever possible. The hBD-2&RBD complexes were solvated with the TIP3P water model in a cubic box under periodic boundary conditions, while maintaining the 1.2 nm minimum distances between the protein atoms and the box boundaries. The net charge of the system was neutralized by adding sufficient chloride ions. Systems were subjected to the steepest-descent minimization for 100 ps, followed by two-staged equilibrations at standard thermo- and barostat (v-rescale-temp for NVT ensembles, 1 ns at 310 K, followed by the Parrinello–Rahman barostat for the NPT ensemble, 5 ns at 1 atm and 310 K). MD simulations were run for 500 ns each (total 124 μs) under the NPT ensemble and particle-mesh Ewald algorithms were used for computing long-range electrostatic interaction.

### Binding Free Energy Parameter Calculations

Binding free energy between hBD-2 (in wild type or spot mutant form) and RBD was calculated by using MM/PBSA program.^24^ Binding free energy was calculated on 100 snapshots extracted during the last 100 ns of the MD simulations. The molecular systems comprising the complex, ligand, and protein can be identified by their estimated binding free energy (Δ*G*) as follows:^25^

1 $${\Delta G}_{\mathrm{bind}}=G_{\mathrm{complex}}-G_{\mathrm{protein}}-G_{\mathrm{ligand}}$$

2 $${\Delta G}_{\mathrm{bind}}=E_{\mathrm{MM}}-G_{\mathrm{sol}}-T\Delta S$$

3 $$E_{MM}=E_{\mathrm{int}}+E_{vdW}+E_{ele}$$

4 $$G_{\mathrm{sol}}=G_{\mathrm{PB}}+G_{\mathrm{SA}}$$

5 $$G_{\mathrm{SA}}=\gamma *\mathrm{SASA}$$

where eq 2 defines *E*_MM_ as the molecular mechanics energy that is the sum of three energetic terms: internal energy (*E*_int_) (where *E*_int_ consists of bond (*E*_bond_), angle (*E*_angle_), dihedral (*E*_dihedral_) energies), van der Waals (*E*_vdW_), and electrostatic (*E*_ele_) energies. The other two terms, *G*_sol_, and −*T*Δ*S*, represent the solvation free energy and the entropy upon hBD-2 binding. For the purpose of obtaining stable results, we utilized the single MD trajectory methodology to calculate the free energy of end-point binding. Eq 4 represents the polar (*G*_PB_) and nonpolar (G_SA_) contributions to the solvation energy, which together make up *G*_sol_. The polar part of the solvation energy can be computed using the PB model, while the nonpolar part can be computed using the solvent-accessible surface area (SASA) and the LCPO algorithm (eq 5).^26^ γ is the surface tension of solvent with the default value of 2.26778 kJ/(mol·nm^2^). Based on several prior computational investigations,^27–30^ the contribution of peptide conformational entropy was disregarded when calculating the binding free energy. Furthermore, the methodology’s specifics are available in studies conducted by Soliman et al.^31^

Further, to monitor the structural deviations of the hBD-2&RBD complexes (wild type and mutants), GROMACS in-house analysis script was used to calculate root-mean-square deviations (RMSD). The number of hydrogen bonds formed between hBD-2 and RBD in both wild type and point mutants was determined using the gmx hbond command in GROMACS. A distance cutoff of 0.35 nm and an angle cutoff of 30° between the donor–acceptor–hydrogen atoms were applied in the hydrogen bonds calculation.

Buried surface area (BSA) analysis was conducted for hBD-2&RBD complexes. The BSA of the complex was determined through a three-step process using the gmx_sasa command from GROMACS.^32^ Initially, the total solvent-accessible surface area of the complex (SASA_complex_) was computed. Following this, the solvent-accessible surface areas of RBD and hBD-2 were individually calculated from the complex trajectory, utilizing a solvent probe size of 0.14 nm.^33^ Finally, the BSA was computed using eq 6.

$$\mathrm{BSA}=0.5*\left({\mathrm{SASA}}_{\mathrm{RBD}}+{\mathrm{SASA}}_{\mathrm{hBD}2}-{\mathrm{SASA}}_{\mathrm{complex}}\right)$$ 6

The heat map graph was plotted (including change of binding free energy and average number of hydrogen bonds formed) using OriginPro software (Version 2024, Origin Lab Corporation, Northampton, MA).

## Results

### Binding Free Energy Analysis

In order to find out which point mutation on hBD-2 can enhance its binding with RBD, the gmx-MM/PBSA program was utilized to compute the binding free energy of each hBD-2 point mutant with RBD based on all-atom simulation trajectories. The average binding free energy (Δ*G*) and its standard deviation for the wild type hBD-2&RBD interaction were predicted to be −40.17 ± 6.17 kcal/mol, consistent with our prior findings.^17^ Additionally, the difference in binding free energy (ΔΔ*G*) between wild-type and mutant systems was calculated using eq 7:

7 $$\Delta \Delta G={\Delta G}_{\mathrm{MT}}-{\Delta G}_{\mathrm{WT}}$$

Here, Δ*G*_MT_ and Δ*G*_WT_ represent the binding free energy changes for the mutant and wild-type systems, respectively. This process was repeated for all 247 hBD-2 mutants interacting with RBD, and the resulting ΔΔ*G* values were used to generate a heat map (Figure 2) (with the ΔΔ*G* values of all 247 mutations of hBD-2 interacting with RBD reported in Tables S1–S13). Analysis of the heat map revealed that most of the point mutations on the targeted region of the hBD-2 weakened the binding of hBD-2 with the RBD. Only 11 point mutations on hBD-2 exhibited negative ΔΔ*G* values (Figure 3), indicating enhanced binding of the hBD-2 point mutant with RBD.

**Figure 2:**
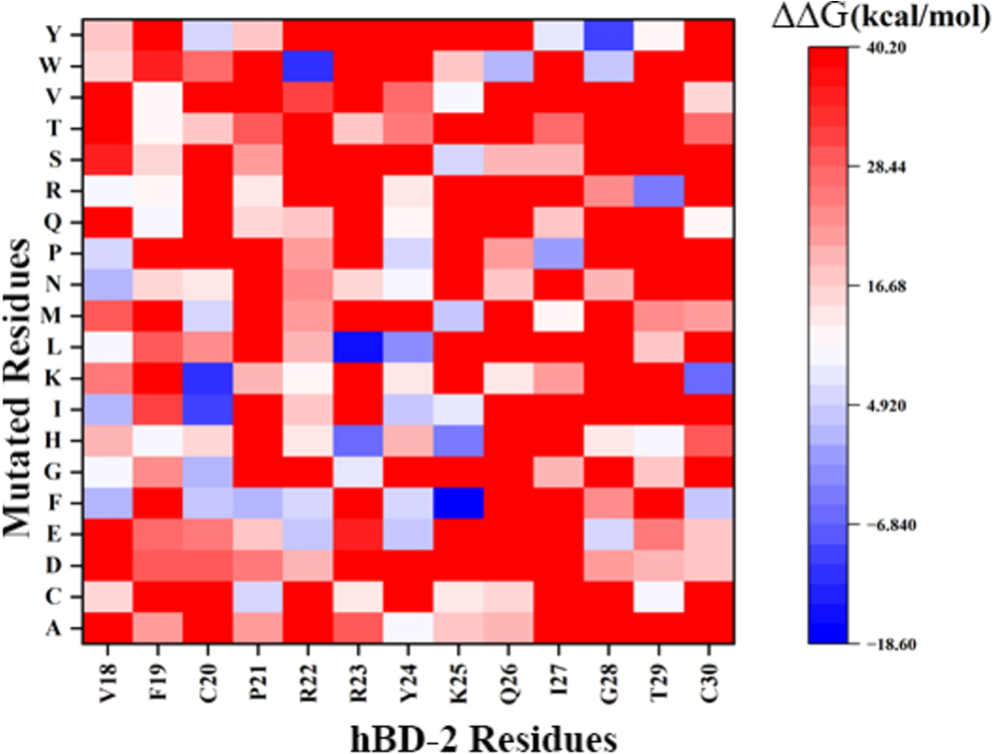
Change of binding interaction energy heat map plot of 247 hBD-2 point mutants binding with RBD.

**Figure 3:**
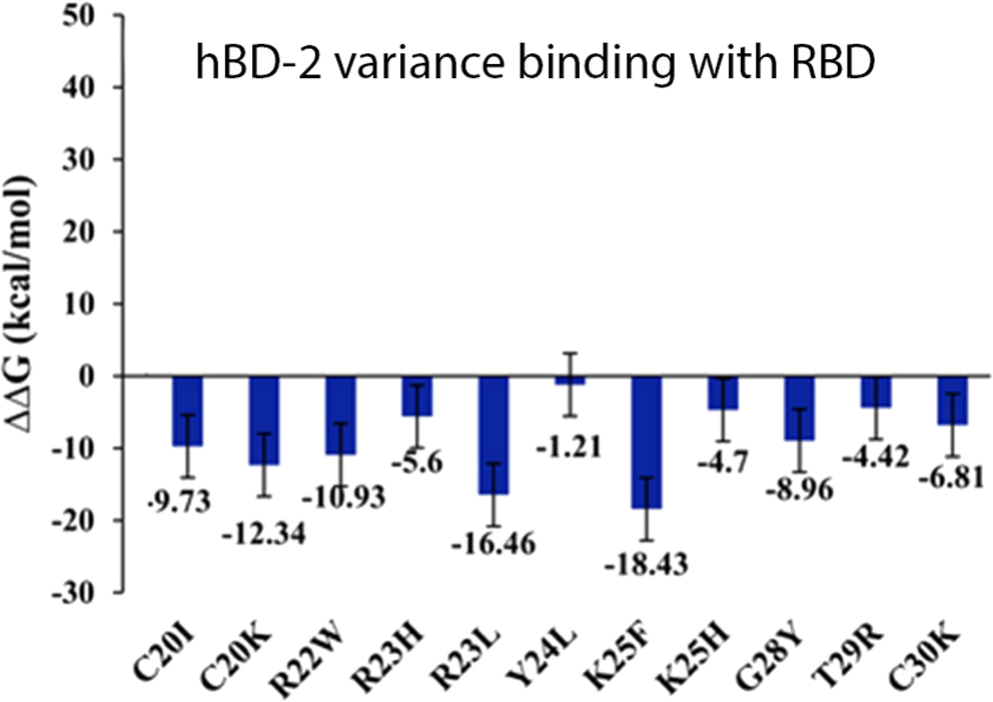
ΔΔ*G* of 11 hBD-2 point mutants binding with RBD which have a binding interaction energy lower than the complex in wild type.

The ΔΔ*G* values of those 11 point mutations are shown in Figure 3. K25F and R23L can significantly enhance the binding of hBD-2 and RBD, while Y24L contributes the least to the binding.

Additionally, the residues implicated in interactions between RBD and hBD-2 in both wild-type and point mutant forms were investigated. Residues participating in interactions during the final 100 ns of MD simulations for the wild-type hBD-2&RBD complex are as follows: G1, I2, G3, T7, K10, S11, F19, I27, G28, T29, and K39 on hBD-2, and Y449, Y453, L455, E484, Q493, Q498, and Y505 on RBD as shown in Figure 4. Specifically, the head (G1, I2), α-helix region (T7), and central region (I27, G28, and T29) of hBD-2 contributed significantly to the binding with RBD, while the tail (K39) of hBD-2 did not. On the other hand, the Y449, L455, E484, Q493, Q498, and Y505 on RBD contributed significantly to the binding with hBD-2 wild type. Checking the charge of residues contributed to the binding, we can see that none of the residues on hBD-2 are charged, and one of six residues on RBD is negatively charged. Thus, the electrostatic interaction is not the major driving force to the binding between hBD-2 and RBD.

**Figure 4:**
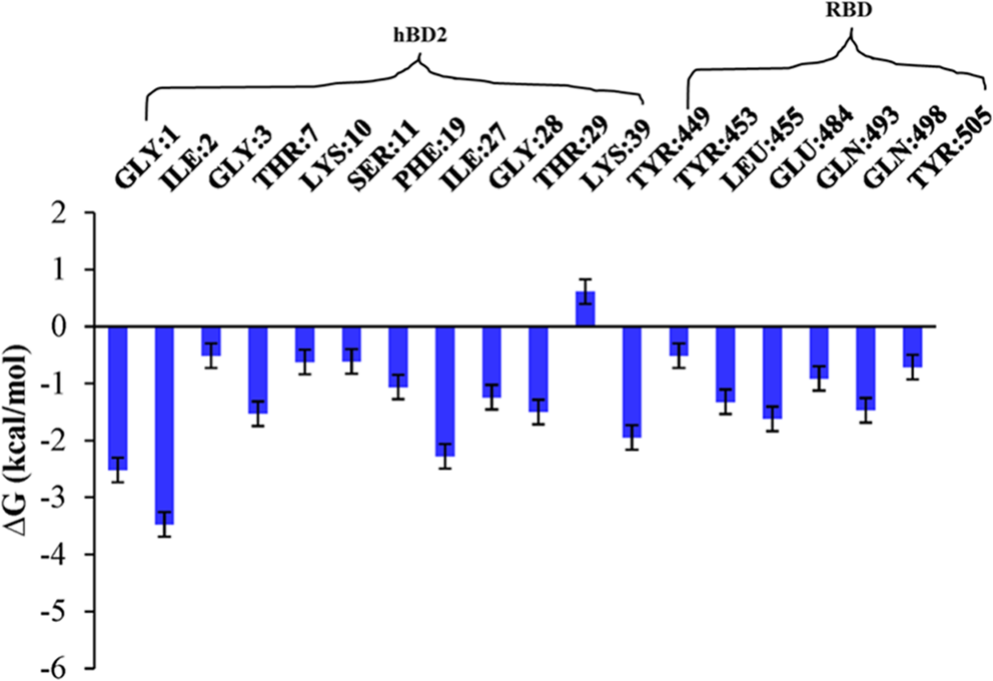
Δ*G* of the residues of wild-type hBD-2&RBD complex involved in interaction during the last 100 ns of the MD simulations.

Similarly, Figure S1 illustrates the residues involved in interactions for mutants C20I, C20K, R22W, R23H, R23L, Y24L, K25F, K25H, G28Y, T29R, and C30K. For mutants C20I and C20K, residues P17, L32, P33, and K36 of hBD-2 exhibit increased contributions to binding free energy (Δ*G*), thereby enhancing the binding with RBD. In R22W mutants, F19, W22, Q26, and I27 of hBD-2 contribute more significantly to Δ*G* compared to the wild-type complex. In R23H and R23L mutants, R22 of hBD-2 contributes the most to Δ*G* value. In the Y24L mutant, L9 is notable, while in K25F, R22 stands out. In K25H, I27 is significant. In G28Y, R22 plays a crucial role. In T29R, I27 is highlighted, and in C30K, K36 of hBD-2 contributes more prominently to binding with RBD compared to the wild type, thus strengthening the interactions. In summary, the point mutants enhanced the binding with RBD through different mechanisms from electrostatic interaction. Most of the residue binding interaction energy profiles of hBD-2 point mutants&RBD are consistent with the wild type, except C30K, T29R, K25F, R23H, and R23L, which weakened the contribution of hBD-2 head to the binding with RBD while enhanced the contribution of hBD-2 central or tail region to the binding. That suggests that point mutation on hBD-2 can affect the binding and interaction between hBD-2 and RBD.

### Structural Stability Analysis

The ongoing structural stability assessment of the hBD-2&RBD complex and its variants has been conducted through RMSD analyses. The average RMSD value for the wild-type hBD-2&RBD complex reaches 0.22 ± 0.03 nm, indicating robust structural stability. Our findings align closely with those of Behairy et al.,^34^ who determined the average RMSD of the wild-type hBD-2&RBD to be 0.25 ± 0.06 nm. The average and standard deviation of RMSD values for the hBD-2 mutants and RBD are provided in Tables S1–S13. Examination of the RMSD values for the hBD-2 mutants bound with RBD reveals that in most cases, when the average RMSD exceeds 0.9 nm, a large structural deviation of the complex from the original binding interface or dissociation of hBD-2 from the RBD happened. Thus, a Δ*G* of zero was assigned to those simulations as shown in Tables S1–S13. Correlating RMSD and Δ*G* based on 247 point-mutation simulations as the original data shown in Tables S1–S13, the result is shown in Figure S2. With RMSD decreasing, log­(−Δ*G*) increases, and thus Δ*G* decreases. Thus, the more stable the complex structure of hBD-2 mutant bound with RBD, the more favorable the binding interaction energy. Structural deviations ranging from 0.2 to 0.6 nm are observed in most mutants exhibiting a Δ*G* higher than the wild type, leading to intermediate deviations but weaker binding.

Some mutants (with average RMSD values between 0.3 and 0.5 nm) stabilize the overall complex structure, and their Δ*G* values are slightly lower than that of the wild type (Table S1 highlighted in blue). Specifically, 11 mutants, C20I, C20K, R22W, R23H, R23L, Y24L, K25H, K25F, G28Y, T29R, and C30K (with RMSD of 0.61 ± 0.20, 0.45 ± 0.06, 0.26 ± 0.03, 0.24 ± 0.03, 0.21 ± 0.03, 0.43 ± 0.10, 0.19 ± 0.02, 0.21 ± 0.02, 0.26 ± 0.03, 0.24 ± 0.02, and 0.23 ± 0.03, respectively), have their time-dependent RMSD plot depicted in Figure 5. RBD can bind with those 11 point mutants stably although C20I and Y23L point mutants initially had large structural deviation from the original structure of the complex in wild type as shown. The alignment of the complex structures of RBD bound with 11 hBD-2 point mutants after 500 ns Gromacs simulations is shown in Figure S3. Those point mutants can bind with RBD at the same binding region as hBD-2 wild type overall.

**Figure 5:**
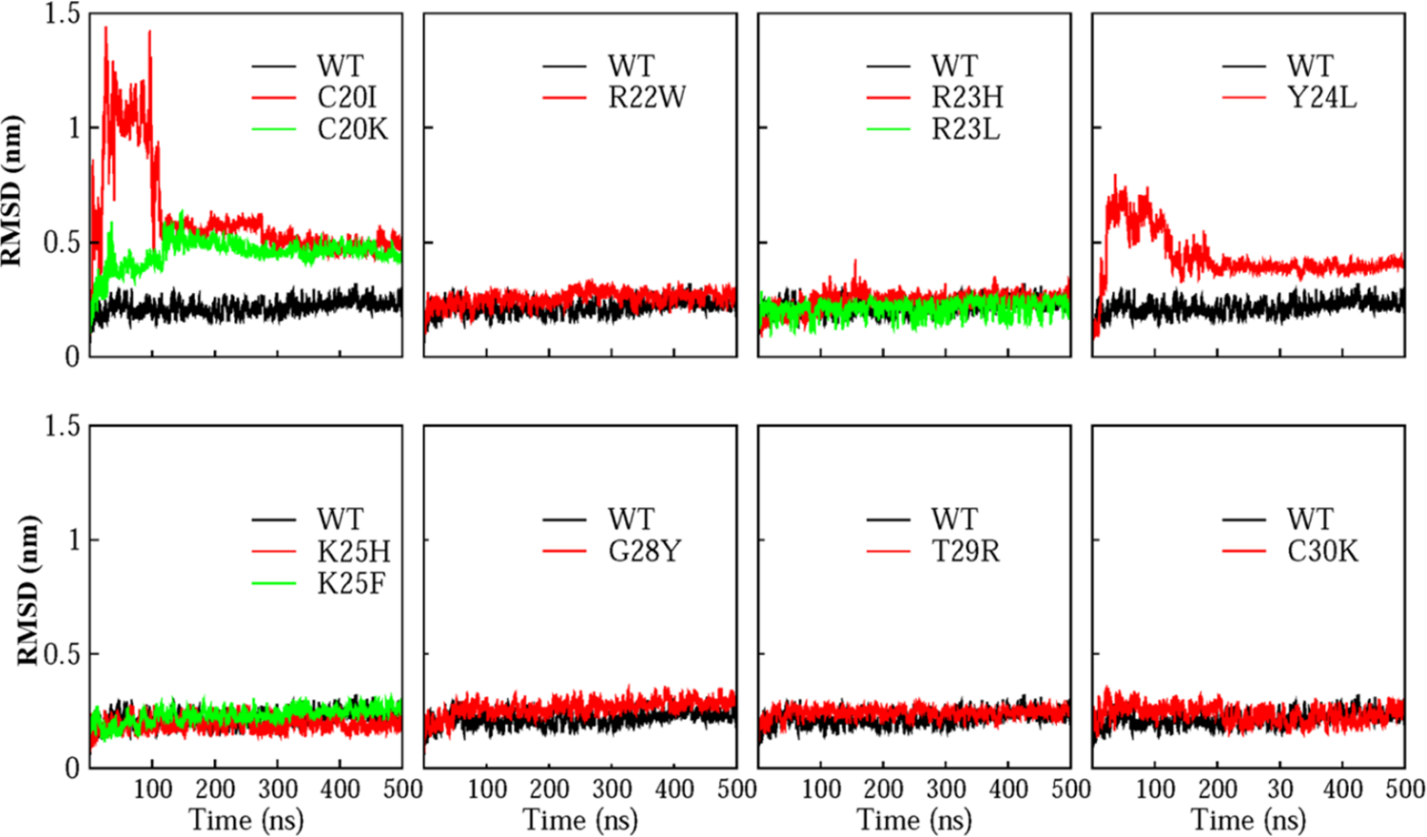
RMSD of the backbone atoms of C20I, C20K, R22W, R23H, R23L, Y24L, K25H, K25F, G28Y, T29R, and C30K mutants of hBD-2&RBD complex. Here, WT is the abbreviation of wild type.

### Hydrogen Bond Analysis, Buried Surface Area

In order to understand the interaction and binding between hBD-2 mutants and RBD, we monitored the hydrogen bond formation between RBD and hBD-2 in wild-type and 247 mutants. Based on the MD simulation trajectories, the average number of hydrogen bonds between RBD and hBD-2 in both wild-type and mutant forms was calculated and is shown in Figure 6 as a heat map. The hBD-2&RBD wild-type complex exhibited an average of 4.62 hydrogen bonds, consistent with our previous study reporting 4 intermolecular hydrogen bonds between hBD-2&RBD.^17^ Figure 6 shows that the average number of hydrogen bonds in point mutants is lower than in the wild type, suggesting destabilization of the hBD-2&RBD complexes by most point mutations.

**Figure 6:**
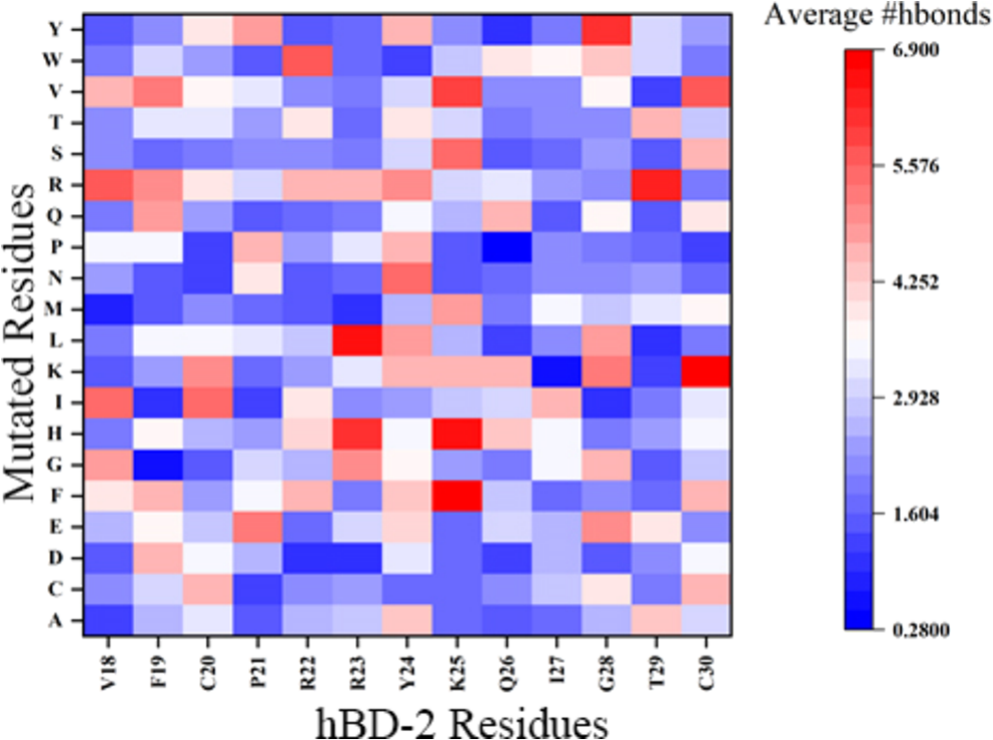
Heat map plot illustrating the average number of hydrogen bonds formed between hBD-2&RBD in wild type and the 247 point mutants over the course of the MD simulations.

However, certain mutants, namely, C20I, C20K, R22W, R23H, R23L, Y24L, K25F, K25H, G28Y, T29R, and C30K, formed a larger number of hydrogen bonds than the wild type: 5.44, 5.03, 5.73, 6.05, 6.55, 4.85, 6.68, 6.55, 6.04, 6.27, and 6.90, respectively. This suggests that these point mutants may actually stabilize the hBD-2&RBD complex more effectively than the wild type by forming a greater number of hydrogen bonds. Those point mutants also have stronger binding with RBD as shown in the binding interaction energy result section. That suggests that forming hydrogen bonds is the major driving force to the binding of hBD-2 with RBD.

To further examine the residues participating in the binding interface of wild-type and specific point mutations (C20I, C20K, R22W, R23H, R23L, Y24L, K25F, K25H, G28Y, T29R, and C30K), the Ligplot program^35^ was utilized on the final complex structures obtained from simulations of both wild-type and point mutants. The results of the wild-type hBD-2&RBD and point mutants K25F and C30K are presented in Figure S4 (as these point mutants have a larger amount of hydrogen bonds compared to others), while the findings for other point mutants such as C20I, C20K, R22W, R23H, R23L, Y24L, K25H, G28Y, and T29R are depicted in Figures S5 and S6. It was found that those 11 point mutations can help to form more hydrogen bonds on the hBD-2 and RBD binding interface than the wild type.

The buried surface area (BSA) between hBD-2 and RBD was calculated as detailed in the Methods section, and the results for the wild-type and point mutants (C20I, C20K, R22W, R23H, R23L, Y24L, K25F, K25H, G28Y, T29R, and C30K) over the course of simulations are plotted in Figure S7. The average BSA data are listed in Table S14. RBD can bind with hBD-2 wild type and 11 point mutants stably during the course of simulations. That aligns with the binding free energy and hydrogen bond analysis results.

## Discussion

hBD-2 is an important part of our body’s defense system. It is a cationic antimicrobial peptide containing three intramolecular disulfide bonds and serves as the body’s initial protection against bacteria and fungi. It is mainly situated in human mucosal surfaces, like the respiratory tract, and plays a role in the innate immune system.^36–38^ hBD-2 can block certain sites that CoV-2 use to enter normal cells.^17^ In this study, we investigated the stability of the hBD-2&RBD complex through MD simulation, concurrently examining the impact of point mutations within the hBD-2 binding region (RES18–30, encompassing a total of 247 mutations) on RBD stability. Our study indicates that most of the point mutations on hBD-2 could not enhance the binding of hBD-2 with RBD, but some specific mutations on hBD-2 can. To design novel hBD-2-based drugs, block mutations that involve mutations on hBD-2 at more than 1 residue site should be tried in the future.

In this study, we employed the gmx-MM/PBSA program to compute the binding free energy changes for RBD&hBD-2 complex in both the wild-type and point mutant forms. Our findings were corroborated by analyses of RMSD, hydrogen bonds, and BSA. The ΔΔ*G* values depicted in Figure 2 revealed that not all point mutations on hBD-2 contribute to the stabilization of the hBD-2&RBD complex. Notably, a subset of 11 mutations, including C20I, C20K, R22W, R23H, R23L, Y24L, K25F, K25H, G28Y, T29R, and C30K, exhibited more negative ΔΔ*G* values, indicative of enhanced interactions between hBD-2&RBD and consequent obstruction of SARS-CoV-2 entry. Specifically, the average binding energies for C20K (−52.51 ± 6.9 kcal/mol), R22W (−51.1 ± 8.7 kcal/mol), R23L (−56.63 ± 6.9 kcal/mol), and K25F (−58.6 ± 8.4 kcal/mol) point mutants were notably lower compared to the average binding energy of the RBD: ACE2 complex (−45 ± 7 kcal/mol).^17^ Thus, those four mutations are highly potential/feasible, which can enhance the binding of hBD-2 and RBD based on our prediction. These observations underscored the heightened efficacy of these point mutants in impeding RBD entry compared to the wild-type hBD-2, which exhibited an average binding energy of −40.17 ± 6.2 kcal/mol.

The RMSD profiles of point mutants C20K, R22W, R23L, and K25F exhibit stabilization based on the result in the last 100 ns of simulations, indicating the complex’s stability. Consistent with these findings, hydrogen bond analysis and BSA results align with the binding free energy outcomes, corroborating the obstruction of RBD entry. Defensins can form stable binding with RBD, or viral surface glycoprotein or chemokine receptor, as demonstrated by several experimental studies,^17,39,40^ to effectively impede viral/microbial infection. To ensure the reliability of our MD simulations, we repeated the simulations for the wild-type and several single-point mutants (C20I, C20K, R22W, R23H, R23L, Y24L, K25F, K25H, G28Y, T29R, and C30K) that exhibited a lower Δ*G* compared to the wild type. We calculated the Δ*G* values for these mutants over the last 100 ns of the repeated simulations and compared them with those of the initial simulations. As shown in Table S15, the Δ*G* values from the repeated simulations are closely aligned with those from the initial simulations, demonstrating the reproducibility of our results. Additionally, we assessed the RMSD (Figure S8), RMSF (Figures S9 and S10), buried surface area (BSA) (Table S16), and average number of hydrogen bonds (Table S17) for these mutants, comparing them with the initial simulations. The consistency between these metrics further confirms the reliability and reproducibility of our simulations. Nevertheless, experimental validation is imperative to ascertain the binding affinity of hBD-2 point mutants with RBD as determined in this study.

## Conclusions

This project involved conducting all-atom molecular dynamics simulations on both the wild type hBD-2&RBD complex and a comprehensive set of point mutants (totaling 247 mutants) of hBD-2 binding with RBD. It was found that although hBD-2 wild type binds with RBD stably, only specific point mutations of hBD-2 enhanced the binding with RBD. Specifically, molecular dynamics simulations illustrated that certain mutations, such as C20I, C20K, R22W, R23H, R23L, Y24L, K25F, K25H, G28Y, T29R, and C30K, led to increased binding stability of the complex, as evidenced by constant RMSD profiles and reduced fluctuations around RBD binding regions. Hydrogen bond analysis further corroborated the formation of stable interactions between those hBD-2 point mutants and RBD, thus the binding of hBD-2 with RBD. These were consistent with binding free energy calculations and BSA analyses as well. In conclusion, our study sheds light on the interaction dynamics between hBD-2 and RBD, revealing significant insights into both wild-type and point mutant complexes. The result can pave the road for designing novel hBD-2-based drugs in the long term.

## Supplementary Material



## Notes

Average Δ*G* and RMSD values of hBD-2 wild-type and spot mutants from V18 to C30 (18 mutants each) bound with RBD, average buried surface area (BSA) for RBD bound with hBD-2 wild-type and 11 spot mutants, Δ*G* and average RMSD and BSA, and the average number of hydrogen bonds formed between hBD-2 wild-type and 11 point mutants binding with RBD based on the original (MD1) and repeat (MD2) simulations; and residues on RBD involved in interactions with hBD-2 wild-type and 11 mutants C20I, C20K, R22W, R23H, R23L, Y24L, K25F, K25H, G28Y, T29R, and C30K (Tables S1–S17); correlation of RMSD and Δ*G* based on 247 hBD-2 point mutants and the wild-type binding with RBD simulation results; alignment of complex structures of RBD bound with 11 hBD-2 point mutants; binding interfaces of RBD bound with hBD-2 wild-type and 11 hBD-2 point mutants; BSA results of the RBD bound with hBD-2 wild-type and 11 point mutants; backbone RMSD plots of the complexes; and RMSF of RBD; RMSF of hBD-2 in wild-type and point mutant systems as a function of time in both MD1 and MD2 simulations (Figures S1–S10) (PDF)
