# Supplementary material for: Exploring the Interaction of RBD with Human β Defensin Type 2 Point Mutants: Insights from Molecular Dynamics Simulations
Source: J Phys Chem B. 2025 Feb 10;129(7):1927–33. doi: 10.1021/acs.jpcb.4c07004 (PMC12422537; doi:10.1021/acs.jpcb.4c07004)
Supplement: Supplementary file 1 [file jp4c07004_si_001.pdf]

Exploring the Interaction of RBD with Human β Defensin Type 2 Point-Mutants: Insights from Molecular Dynamics Simulations

Ishrat Jahan, Liqun Zhang

Chemical Engineering Department, University of Rhode Island, Kingston, RI, 02881

Tables

Table S1. The average ΔG and RMSD and standard deviations of RBD bound with hBD-2 wildtype and V18 mutants.

V18 mutants	ΔG (kcal/mol) ± SEM	RMSD (nm) ± SD
Native	-40.17 ± 0.62	0.22± 0.03
V18A	0	1.08 ± 0.20
V18L	-31.30 ± 0.85	0.39 ± 0.07
V18I	-39.07 ± 0.66	0.20 ± 0.03
V18M	-10.76 ± 1.00	0.47 ± 0.28
V18F	-38.86 ± 1.41	0.48 ±0.09
V18Y	-23.01 ± 0.57	0.72 ± 0.39
V18W	-24.41 ± 0.56	0.87 ± 0.37
V18T	0	1.06 ± 0.14
V18S	-3.92± 0.63	1.10 ± 0.29
V18R	-30.50 ± 1.00	0.22 ±0.03
V18C	-24.05 ± 1.02	0.46 ± 0.11
V18D	0	1.01 ± 0.30
V18E	0	1.08 ± 0.26
V18G	-29.96 ± 0.88	0.26 ±0.04
V18H	-21.22 ± 0.46	0.77 ± 0.39
V18K	-15.17 ±0.73	0.60 ± 0.37
V18N	-37.25± 0.4	0.50 ± 0.09
V18P	-34.53 ± 0.6	0.47 ± 0.12
V18Q	0	1.18 ± 0.25

Table S2. The average ΔG and RMSD and standard deviations of RBD bound with hBD-2 wildtype and F19 mutants.

F19 mutants	ΔG (kcal/mol) ± SEM	RMSD (nm) ± SD
Native	-40.17 ± 0.62	0.22± 0.03
F19A	-17.85 ± 0.59	0.78 ±0.44
F19I	-9.43 ± 0.70	0.88 ± 0.20
F19L	-9.89 ± 0.88	0.39 ± 0.27
F19M	0	1.03 ± 0.22
F19Y	0	1.05 ± 0.15
F19W	-4.20 ± 0.8	0.52 ± 0.36
F19V	-27.88 ± 0.73	0.20 ± 0.02
F19C	0.01± 0.01	1.08 ± 0.16
F19D	-11.57 ± 0.88	0.55 ± 0.48
F19E	-11.92 ± 0.74	0.31 ± 0.17
F19G	-16.32 ± 0.68	0.71 ± 0.24
F19H	-31.05 ± 0.98	0.32 ± 0.04
F19K	0	1.10 ± 0.27
F19S	-23.89 ± 0.67	0.50 ± 0.05
F19P	0	1.14 ± 0.18
F19Q	-31.04 ± 0.83	0.21 ±0.03
F19R	-28.20 ± 0.76	0.19 ±0.02
F19T	-27.55 ± 1.09	0.29 ±0.08
F19N	-24.67 ± 0.43	0.49 ± 0.05

Table S3. The average ΔG and RMSD and standard deviations of RBD bound with hBD-2 wildtype and C20 mutants

C20 mutants	ΔG (kcal/mol) ± SEM	RMSD (nm) ± SD
Native	-40.17 ± 0.62	0.22± 0.03
C20G	-37.86 ± 0.67	0.75 ± 0.15
C20P	0	1.22 ± 0.17
C20D	-9.99 ± 0.35	0.26 ± 0.15
C20N	-25.52 ± 1.03	0.37 ±0.07
C20A	0	1.22 ±0.16
C20L	-16.91 ± 0.7	0.23 ± 0.17
C20K	-52.51 ± 0.69	0.45 ± 0.06
C20M	-34.17 ± 0.5	0.49 ± 0.05
C20H	-24.79 ± 0.86	0.42 ±0.08
C20Q	-0.18 ± 0.33	0.69 ± 0.26
C20F	-36.43 ± 1.03	0.55 ± 0.35
C20T	-23.05 ± 0.76	0.90 ± 0.14
C20V	0	1.03 ± 0.36
C20W	-12.49 ± 0.5	0.65 ± 0.19
C20Y	-33.9 ± 0.74	0.48 ± 0.06
C20R	0	1.25 ± 0.14
C20E	-15.02 ± 0.81	0.39 ± 0.15
C20S	0	1.13 ± 0.13
C20I	-49.9 ± 0.97	0.61 ± 0.20

Table S4. The average ΔG and RMSD and standard deviations of RBD bound with hBD-2 wildtype and P21 mutants.

**P21 mutants**	**ΔG (kcal/mol) ± SEM**	**RMSD (nm) ± SD**
Native	-40.17 ± 0.62	0.22± 0.03
P21A	-19.23 ± 0.31	0.64 ± 0.21
P21I	0	0.80 ± 0.41
P21L	0	1.16 ± 0.17
P21C	-34.07 ± 0.59	0.44 ± 0.08
P21G	0	1.10 ± 0.27
P21R	-26.37 ± 0.54	0.44 ± 0.12
P21N	0	1.07 ± 0.20
P21D	-15.04 ± 0.58	1.13 ± 0.20
P21E	-22.64 ± 1.0	0.24 ± 0.06
P21Q	-24.94 ± 0.69	0.53 ± 0.05
P21H	0	1.08 ± 0.38
P21K	-20.97 ± 0.68	0.94 ± 0.29
P21M	0	1.01 ± 0.18
P21F	-38.24 ± 0.37	0.36 ± 0.05
P21S	-19.51 ± 0.71	0.94 ± 0.29
P21T	-11.71 ± 1.03	0.57 ± 0.39
P21W	0	0.98 ± 0.38
P21Y	-22.31 ± 0.92	0.25 ± 0.15
P21V	0	1.06 ± 0.17

Table S5. The average ΔG and RMSD and standard deviations of RBD bound with hBD-2 wildtype and R22 mutants.

**R22 mutants**	**ΔG (kcal/mol) ± SEM**	**RMSD (nm) ± SD**
Native	-40.17 ± 0.62	0.22± 0.03
R22H	-26.39 ± 0.52	0.33 ± 0.03
R22K	-28.15 ± 0.45	0.60 ± 0.37
R22A	0	0.89 ± 0.40
R22N	-16.17 ± 0.45	0.45 ± 0.10
R22D	-20.48 ± 0.73	0.47 ± 0.15
R22C	0	1.03 ± 0.12
R22E	-35.28 ± 1.40	0.42 ± 0.06
R22Q	-22.71 ± 0.68	0.45 ± 0.07
R22G	0	1.09 ± 0.31
R22I	-22.96 ± 1.06	0.20 ± 0.03
R22L	-20.38 ± 0.58	0.39 ± 0.17
R22M	-17.69 ± 0.51	0.85 ± 0.34
R22F	-34.4 ± 0.66	0.26 ± 0.04
R22P	-19.4 ± 0.44	0.56 ± 0.22
R22S	0	1.23 ± 0.23
R22T	0	1.07 ± 0.31
R22W	-51.1 ± 0.87	0.26 ± 0.03
R22Y	0	0.85 ± 0.23
R22V	-8.37 ± 0.83	0.49 ± 0.33

Table S6. The average ΔG and RMSD and standard deviations of RBD bound with hBD-2 wildtype and R23 mutants.

**R23 mutants**	**ΔG (kcal/mol) ± SEM**	**RMSD (nm) ± SD**
Native	-40.17 ± 0.62	0.22± 0.03
R23H	-45.77 ± 0.75	0.24 ± 0.03
R23K	0	1.13 ± 0.20
R23A	-11.02 ± 0.54	0.35 ± 0.11
R23N	-24.81 ± 0.93	0.46 ± 0.10
R23D	0	1.10 ± 0.14
R23C	-25.9 ± 0.7	0.47 ± 0.19
R23E	-4.07 ± 0.60	0.41 ± 0.26
R23Q	0	1.21 ± 0.14
R23G	-32.77 ± 1.01	0.20 ± 0.02
R23I	0	0.87 ± 0.41
R23L	-56.63 ± 0.69	0.21 ± 0.03
R23M	0	0.94 ± 0.24
R23F	0	0.90 ± 0.25
R23P	0	1.01 ± 0.10
R23S	0	0.68 ± 0.37
R23T	-22.69 ± 0.93	0.39 ± 0.13
R23W	0	1.13 ± 0.18
R23Y	0	0.73 ± 0.27
R23V	0	0.68 ± 0.40

Table S7. The average ΔG and RMSD and standard deviations of RBD bound with hBD-2 wildtype and Y24 mutants.

Y24 mutants	ΔG (kcal/mol) ± SEM	RMSD (nm) ± SD
Native	-40.17 ± 0.62	0.22 ± 0.03
Y24A	-30.12 ± 0.63	0.24 ± 0.03
Y24F	-33.34 ± 1.07	0.27 ± 0.17
Y24L	-41.38 ± 0.57	0.43 ± 0.10
Y24R	-26.88 ± 0.7	0.18 ± 0.03
Y24N	-30.13 ± 0.59	0.25 ± 0.02
Y24D	0	1.08 ± 0.27
Y24C	0	0.81 ± 0.47
Y24E	-36.37 ± 0.51	0.26 ± 0.06
Y24Q	-28.1 ± 0.64	0.35 ± 0.14
Y24G	0	1.10 ± 0.33
Y24H	-21.29 ± 0.83	0.27 ± 0.04
Y24I	-36.56 ± 0.82	0.45 ± 0.10
Y24K	-26.47 ± 0.78	0.21 ± 0.03
Y24M	0	0.76 ± 0.34
Y24P	-34.75 ± 0.69	0.21 ± 0.04
Y24S	0	1.21 ± 0.21
Y24T	-14.2 ± 0.72	0.60 ± 0.37
Y24W	0	0.85 ± 0.14
Y24V	-12.62 ± 0.79	0.27 ± 0.04

Table S8. The average ΔG and RMSD and standard deviations of RBD bound with hBD-2 wildtype and K25 mutants.

K25 mutants± SEM	ΔG (kcal/mol) ± SEM	RMSD (nm) ± SD
Native	-40.17 ± 0.62	0.22± 0.03
K25H	-44.87 ± 0.82	0.19 ± 0.02
K25R	0	0.87 ± 0.44
K25A	-22.99 ± 0.52	0.52 ± 0.22
K25N	0	0.63 ± 0.23
K25D	0	0.63 ± 0.43
K25C	-25.91 ± 0.65	0.46 ± 0.15
K25E	0	0.62 ± 0.38
K25Q	0	0.91 ± 0.39
K25G	0	0.99 ± 0.38
K25I	-32.79 ± 0.52	0.31 ± 0.09
K25L	0	0.95 ± 0.25
K25M	-36.81 ± 0.6	0.21 ± 0.03
K25F	-58.6 ± 0.84	0.21 ± 0.02
K25P	0	1.01 ± 0.22
K25S	-33.89 ± 0.7	0.18 ± 0.02
K25T	0	0.92 ± 0.44
K25W	-21.78 ± 0.5	0.27 ± 0.04
K25Y	0	1.02 ± 0.20
K25V	-30.3 ± 0.95	0.29 ± 0.02

Table S9. The average ΔG and RMSD and standard deviations of RBD bound with hBD-2 wildtype and Q26 mutants.

Q26 mutants	ΔG (kcal/mol) ± SEM	RMSD (nm) ± SD
Native	-40.17 ± 0.62	0.22± 0.03
Q26A	-20.21 ± 0.78	0.47± 0.08
Q26R	0	1.1 ± 0.31
Q26N	-23.26 ± 0.43	0.44 ± 0.05
Q26D	0	0.96 ± 0.31
Q26C	-23.9 ± 0.8	0.55 ± 0.20
Q26E	0	1.42 ± 0.20
Q26G	0	0.98 ± 0.30
Q26H	0	0.86 ± 0.45
Q26I	0	1.07 ± 0.30
Q26L	0	1.1 ± 0.33
Q26K	-27.23 ± 0.59	0.19 ± 0.03
Q26M	0	0.51 ±0.30
Q26F	0	1.06 ± 0.17
Q26P	-17.96 ± 0.33	0.57 ± 0.16
Q26S	-20.36 ± 0.57	0.46 ± 0.11
Q26T	0	1.05 ± 0.28
Q26W	-37.9 ± 0.64	0.49 ± 0.28
Q26Y	0	0.95 ± 0.11
Q26V	0	1.02 ± 0.24

Table S10. The average ΔG and RMSD and standard deviations of RBD bound with hBD-2 wildtype and I27 mutants.

I27 mutants	ΔG (kcal/mol) ± SEM	RMSD (nm) ± SD
Native	-40.17 ± 0.62	0.22± 0.03
I27A	0	0.92 ± 0.12
I27R	0	0.82 ± 0.35
I27N	0	0.94 ± 0.12
I27D	0	0.89 ± 0.40
I27C	0	0.78 ± 0.29
I27E	0	1.01 ± 0.10
I27Q	-22.93 ± 0.73	0.45 ± 0.09
I27G	-21.16 ± 0.62	0.57 ± 0.10
I27H	0	0.94 ± 0.39
I27L	0	1.07 ± 0.23
I27K	-18.41 ± 0.38	0.54 ± 0.06
I27M	-28.81 ± 0.56	0.57 ± 0.09
I27F	0	0.93 ± 0.12
I27P	-39.81 ± 0.57	0.33 ± 0.05
I27S	-20.01 ± 0.83	0.51 ± 0.08
I27T	-13.46 ± 0.51	0.59 ± 0.31
I27W	0	0.69 ± 0.09
I27Y	-32.05 ± 0.67	0.43 ± 0.16
I27V	0	1.00 ± 0.26

Table S11. The average ΔG and RMSD and standard deviations of RBD bound with hBD-2 wildtype and G28 mutants.

G28 mutants	ΔG (kcal/mol) ± SEM	RMSD (nm) ± SD
Native	-40.17 ± 0.62	0.22± 0.03
G28A	0	1.16 ± 0.18
G28R	-16.5 ± 0.27	0.41 ± 0.09
G28N	-19.89 ± 0.54	0.37 ± 0.09
G28D	-19.44 ± 0.85	0.67 ± 0.22
G28C	0	0.66 ± 0.45
G28E	-34.03 ± 0.63	0.19 ± 0.03
G28Q	0	1.19 ± 0.20
G28H	-25.63 ± 0.7	0.40 ± 0.04
G28I	0	0.92 ± 0.38
G28L	0	1.10 ± 0.15
G28K	0	0.89 ± 0.06
G28M	0	1.06 ± 0.26
G28F	-16.89 ± 0.72	0.47 ± 0.04
G28P	0	0.67 ± 0.26
G28S	0	0.95 ± 0.33
G28T	0	0.85 ± 0.40
G28W	-36.73 ± 0.79	0.23 ± 0.04
G28Y	-49.13± 1.16	0.26 ± 0.03
G28V	0	0.97 ± 0.15

Table S12. The average ΔG and RMSD and standard deviations of RBD bound with hBD-2 wildtype and T29 mutants.

T29 mutants	ΔG (kcal/mol) ± SEM	RMSD (nm) ± SD
Native	-40.17 ± 0.62	0.22± 0.03
T29A	0	1.16 ± 0.12
T29R	-44.59 ± 0.61	0.24 ± 0.02
T29N	0	1.11± 0.24
T29D	-20.35 ± 0.51	0.42 ± 0.10
T29C	-30.76 ± 0.46	0.51 ± 0.09
T29E	-14.06 ± 0.68	0.38 ± 0.16
T29Q	0	1.05 ± 0.16
T29H	-30.84 ± 0.52	0.41 ± 0.04
T29I	0	1.09 ± 0.34
T29L	-21.75 ± 0.65	0.43 ± 0.05
T29K	0	0.85 ± 0.19
T29M	-17.4 ± 0.66	0.37± 0.18
T29F	0	0.91 ± 0.23
T29P	0	1.11 ± 0.22
T29S	0	1.18 ± 0.28
T29G	-23.05 ± 0.5	0.43 ± 0.05
T29W	0	0.85 ± 0.41
T29Y	-29.13 ± 1.34	0.35 ± 0.04
T29V	0	1.05 ± 0.24

Table S13. The average ΔG and RMSD and standard deviations of RBD bound with hBD-2 wildtype and C30 mutants.

**C30 mutants**	**ΔG (kcal/mol) ± SEM**	**RMSD (nm) ± SD**
Native	-40.17 ± 0.62	0.22± 0.03
C30A	0	1.29 ± 0.09
C30R	0	1.11 ± 0.13
C30N	0	0.87± 0.29
C30D	-22.94 ± 0.87	0.36 ± 0.05
C30T	-13.35 ± 0.65	0.38 ± 0.18
C30E	0	0.61± 0.14
C30Q	-27.75 ± 0.41	0.31 ± 0.09
C30H	-10.48 ± 0.71	0.54 ± 0.41
C30I	0	0.75 ± 0.39
C30L	0	0.99 ± 0.11
C30K	-46.98 ± 0.91	0.23± 0.03
C30M	-17.74 ± 1.07	0.24 ± 0.06
C30F	-35.92 ± 0.94	0.25± 0.06
C30P	0	0.97 ± 0.23
C30S	0	1.13 ± 0.18
C30G	0	0.97 ± 0.36
C30W	0	0.84 ± 0.28
C30Y	0	0.70± 0.21
C30V	-25.21 ± 0.7	0.32 ± 0.03

Table S14. The average buried surface area (BSA) of RBD bound with hBD-2 wildtype and 11 spot-mutants.

**Mutation**	BSA(nm^2)	**Mutation**	BSA(nm^2)
**Wildtype**	7.23	**Y24L**	5.98
**C20I**	6.07	**K25F**	7.77
**C20K**	7.66	**K25H**	7.96
**R22W**	8.69	**G28Y**	8.19
**R23H**	8.05	**T29R**	8.32
**R23L**	7.76	**C30K**	9.03

Table S15. The ΔG (kcal/mol) and average RMSD (Å) of hBD-2 wildtype and 11 point-mutants binding with RBD based on the original (MD1) and repeat (MD2) simulations.

Point mutants	ΔG (kcal/mol) MD1	ΔG (kcal/mol) MD2	RMSD (Å) MD1	RMSD (Å) MD2
Native	-40.17	-38.99	2.2	3.6
C20K	-52.51	-53.45	4.5	3.2
C20I	-49.90	-46.82	6.1	4.1
R22W	-51.1	-49.50	2.6	2.3
R23H	-45.77	-46.40	2.4	1.2
R23L	-56.63	-56.55	2.1	2.2
Y24L	-41.38	-37.89	4.3	3.9
K25H	-44.87	-39.89	1.9	2.2
K25F	-58.6	-57.61	2.1	1.9
G28Y	-49.13	-44.90	2.6	5.5
T29R	-44.59	-40.65	2.4	4.0
C30K	-46.98	-39.56	2.3	3.0

Table S16: The BSA of the hBD-2 wildtype and 11 point-mutants bound with RBD in both MD1 and MD2 simulations.

Point mutants	BSA (Å^2) MD1	BSA (Å^2) MD2
Native	723.6788	583.2201
C20I	766.3318	879.5224
C20K	607.6718	359.5594
R22W	869.2999	757.7319
R23H	805.1625	600.2382
R23L	776.3946	848.7081
Y24L	598.8601	416.2745
K25F	781.2726	719.3856
K25H	776.9052	791.9818
G28Y	819.8145	612.7441
T29R	831.8737	517.1486
C30K	903.3599	831.6013

Table S17: The average number of hydrogen bonds formed between RBD and hBD-2 wildtype and 11 point-mutants during MD1 and MD2 simulations.

Point mutants	No. of H-bonds (MD1)	No. of H-bonds (MD2)
Native	4.62	3.05
C20I	5.44	4.14
C20K	5.03	4.08
R22W	5.73	4.43
R23H	6.05	4.73
R23L	6.55	4.84
Y24L	4.85	3.80
K25F	6.68	4.43
K25H	6.55	4.18
G28Y	6.04	3.76
T29R	6.27	3.94
C30K	6.90	3.85

Figures

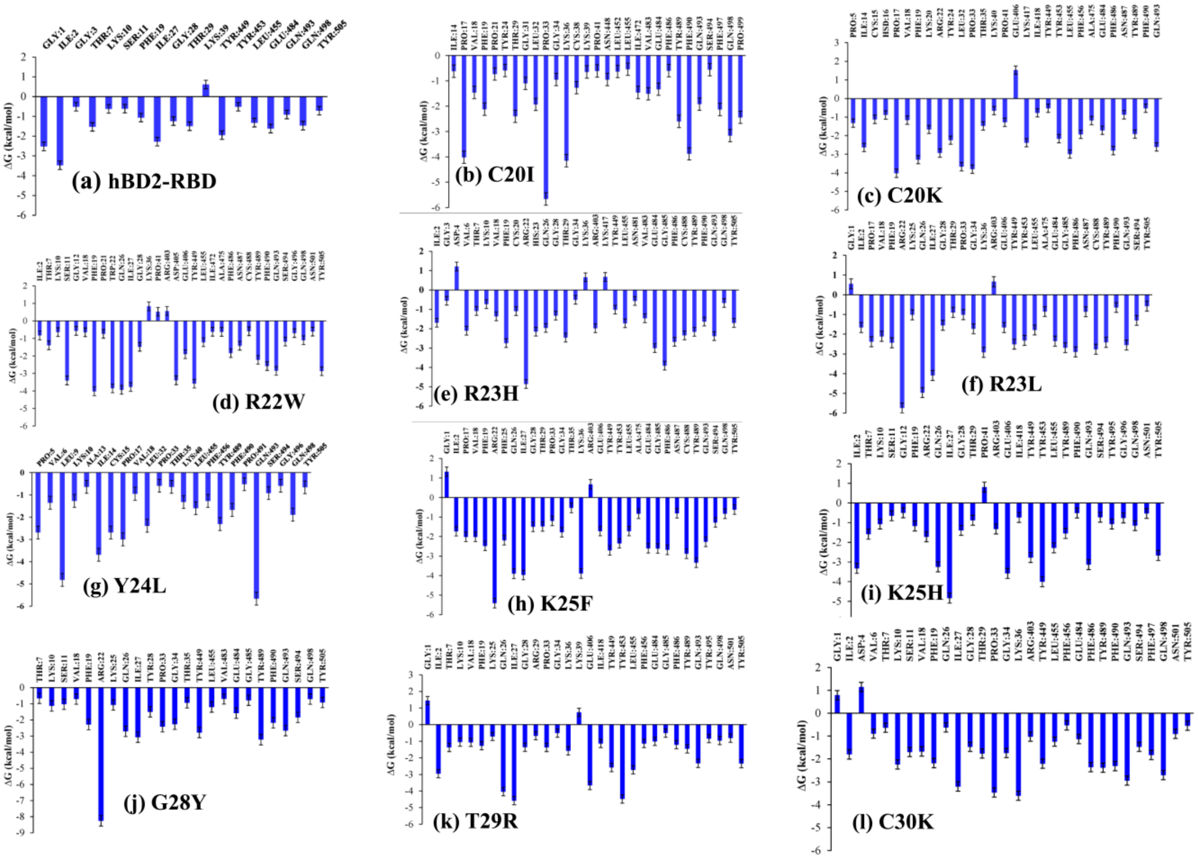

Figure S1. The ΔG between residues on RBD and hBD-2 mutants C20I, C20K, R22W, R23H, R23L, Y24L, K25F, K25H, G28Y, T29R, and C30K along with hBD-2 wildtype.

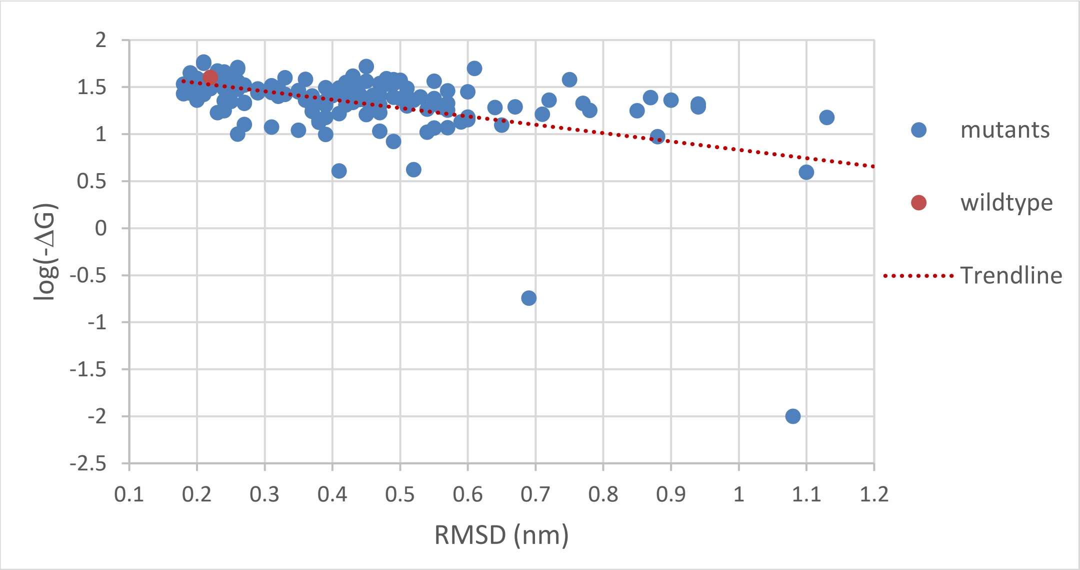

Figure S2. Correlation of RMSD (nm) and ΔG (kcal/mol) based on 247 hBD-2 point-mutants and the wildtype binding with RBD simulation results. All the data with ΔG=0 was not included.

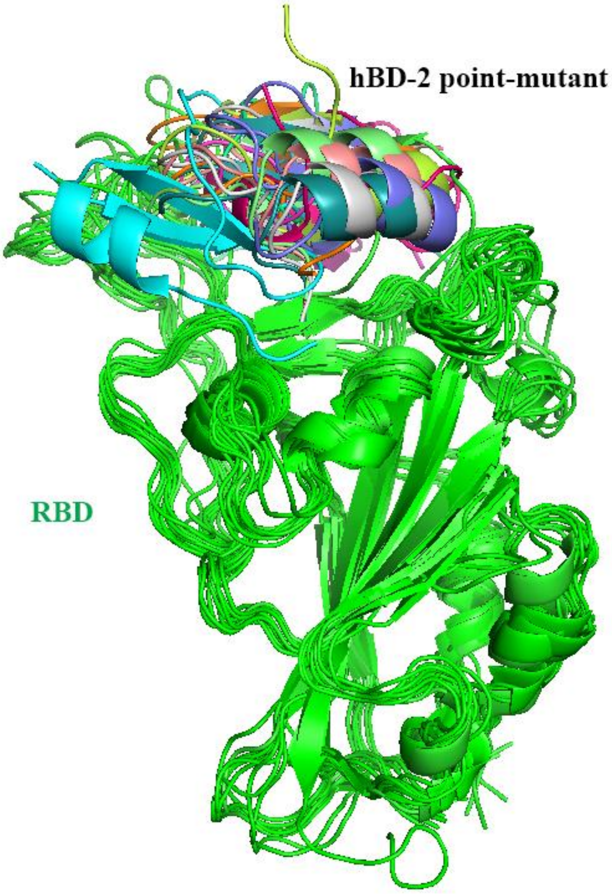

Figure S3. The alignment of complex structures of RBD (in green) bound with 11 hBD-2 point-mutants (C20I, C20K, R22W, R23H, R23L, Y24L, K25F, K25H, G28Y, T29R, C30K) after 500 ns all-atom Gromacs simulations**.**

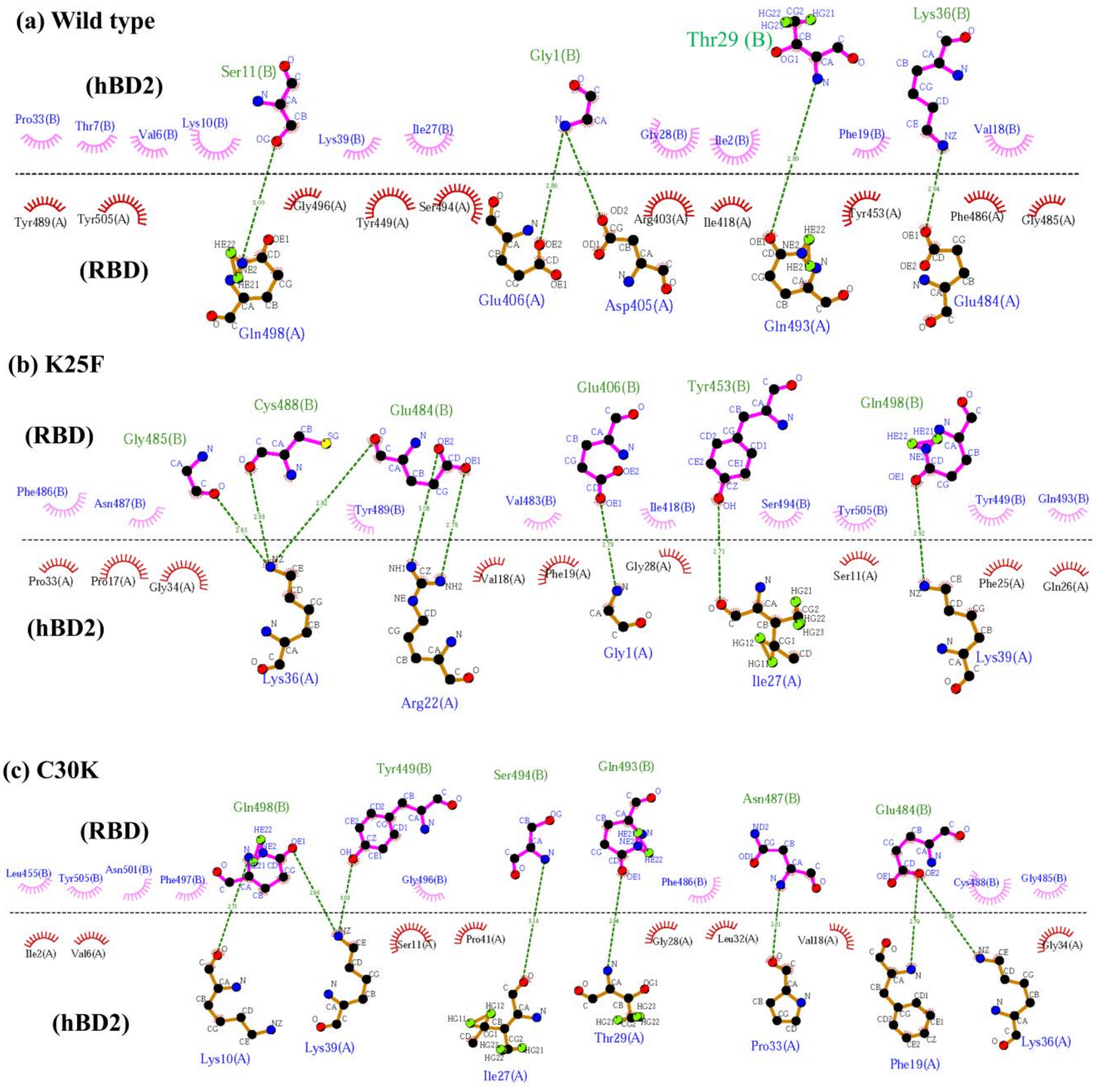

Figure S4. The binding interfaces of complexes of RBD bound with hBD-2 wildtype (a), hBD-2 K25F mutant (b), hBD-2 C30K mutant (c), which were generated using the Ligplot program. Residues engaged in hydrogen bonds are linked by dashed green lines, while those participating in hydrophobic interactions are depicted in arcs.

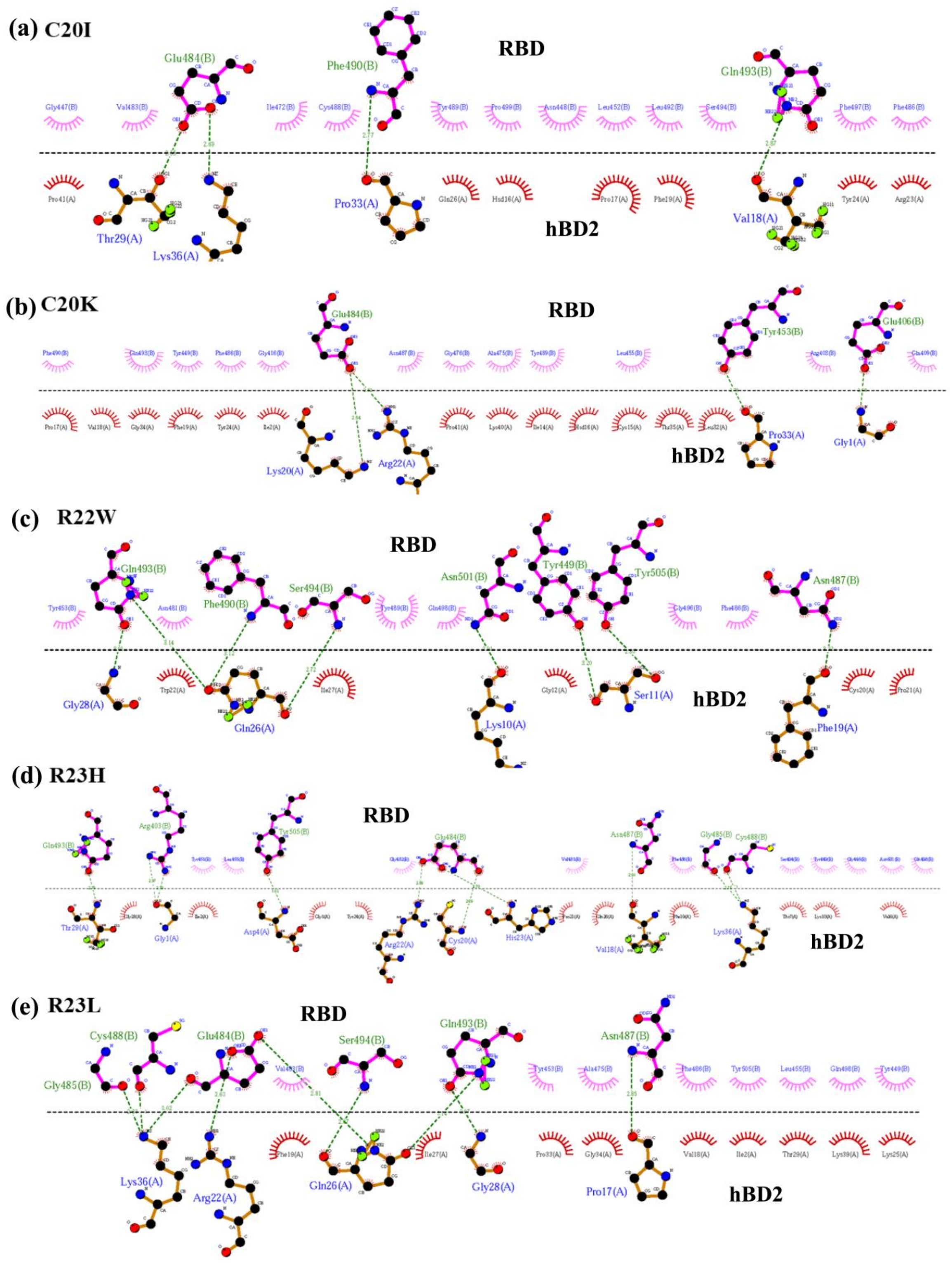

Figure S5. The binding interfaces of complexes of RBD bound with hBD-2 C20I (a), C20K(b), R22W(c), R23H(d), R23L(e), which were generated using the Ligplot program. Residues engaged in hydrogen bonds are linked by dashed green lines, while those participating in hydrophobic interactions are depicted in arcs.

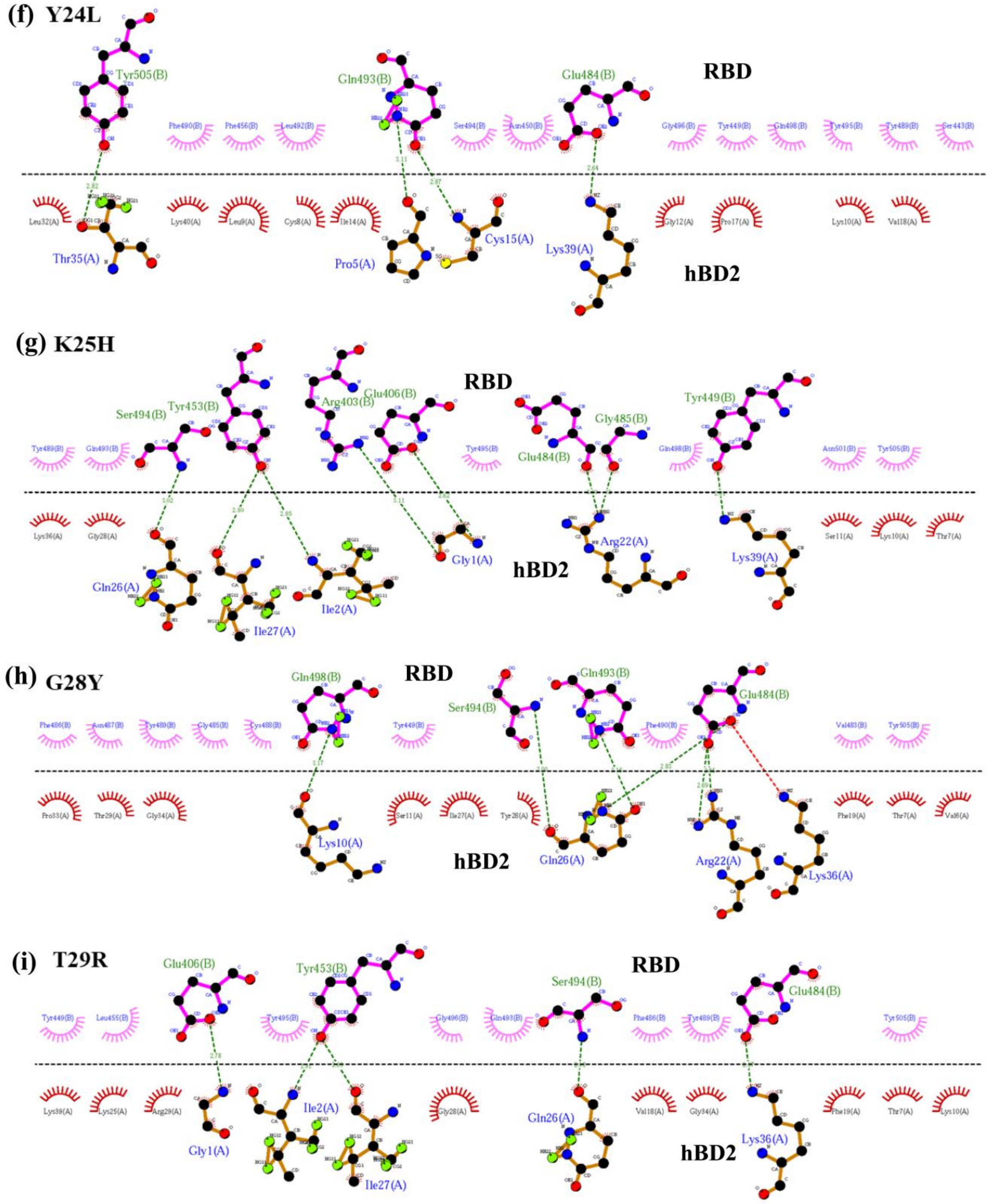

Figure S6. The binding interfaces of complexes of RBD bound with hBD-2 Y24L(a), K25H(b), G28Y(c), and T29R(d), which were generated using the Ligplot program. Residues engaged in hydrogen bonds are linked by dashed green lines, while those participating in hydrophobic interactions are depicted in arcs.

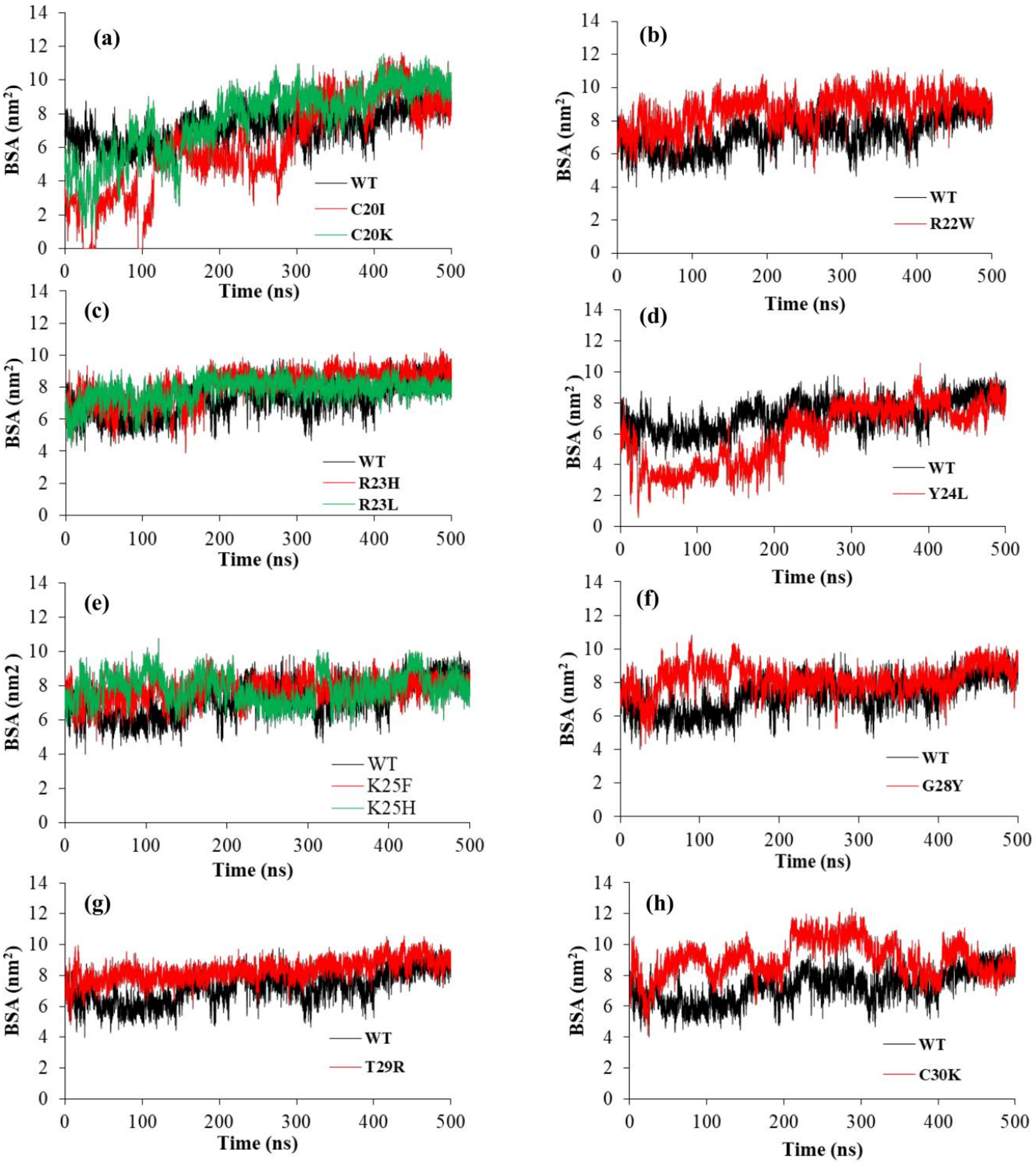

Figure S7. The buried surface area (BSA) results of the RBD bound with hBD-2 wildtype and 11 point-mutants (C20I, C20K, R22W, R23H, R23L, Y24L, K25F, K25H, G28Y, T29R, and C30K) during 500 ns MD simulations.

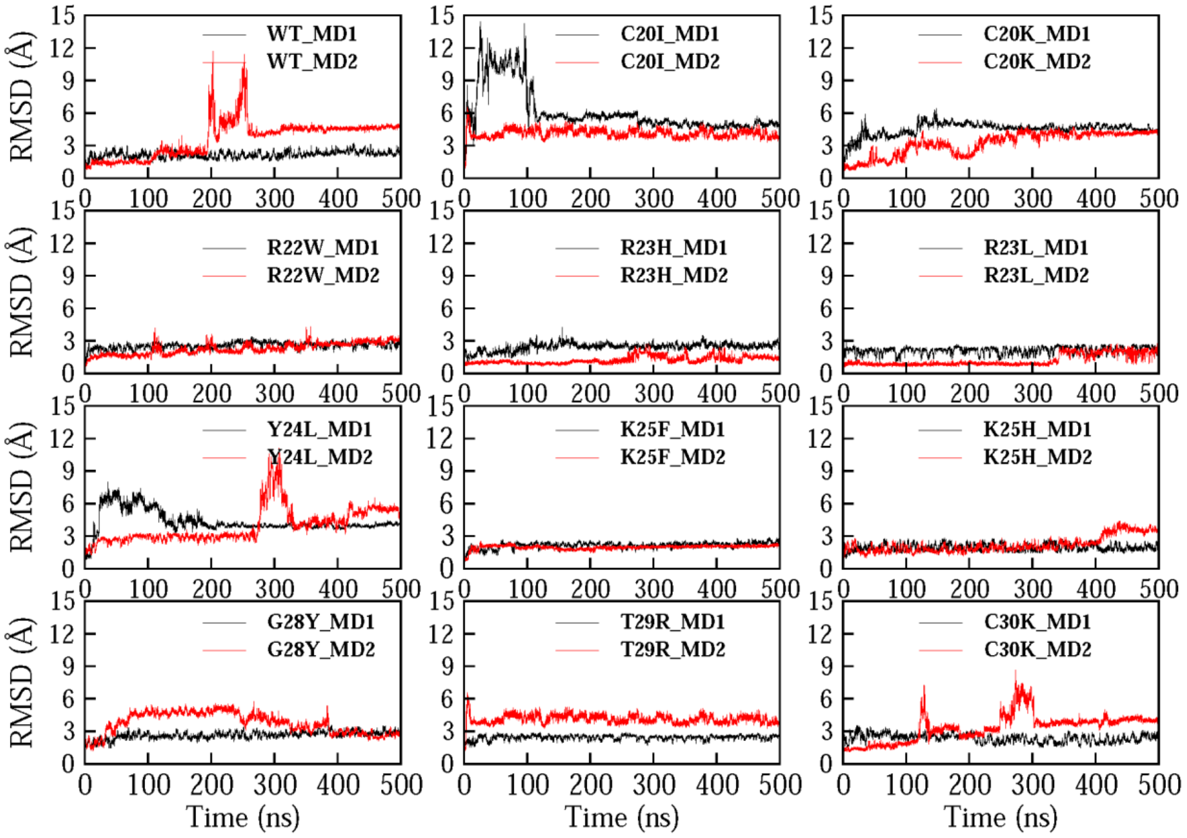

Figure S8: The backbone RMSD plots of the complexes of RBD bound with hBD-2 wildtype and 11 point-mutants as a function of simulation time in both MD1(in black) and MD2 (in red) simulations.

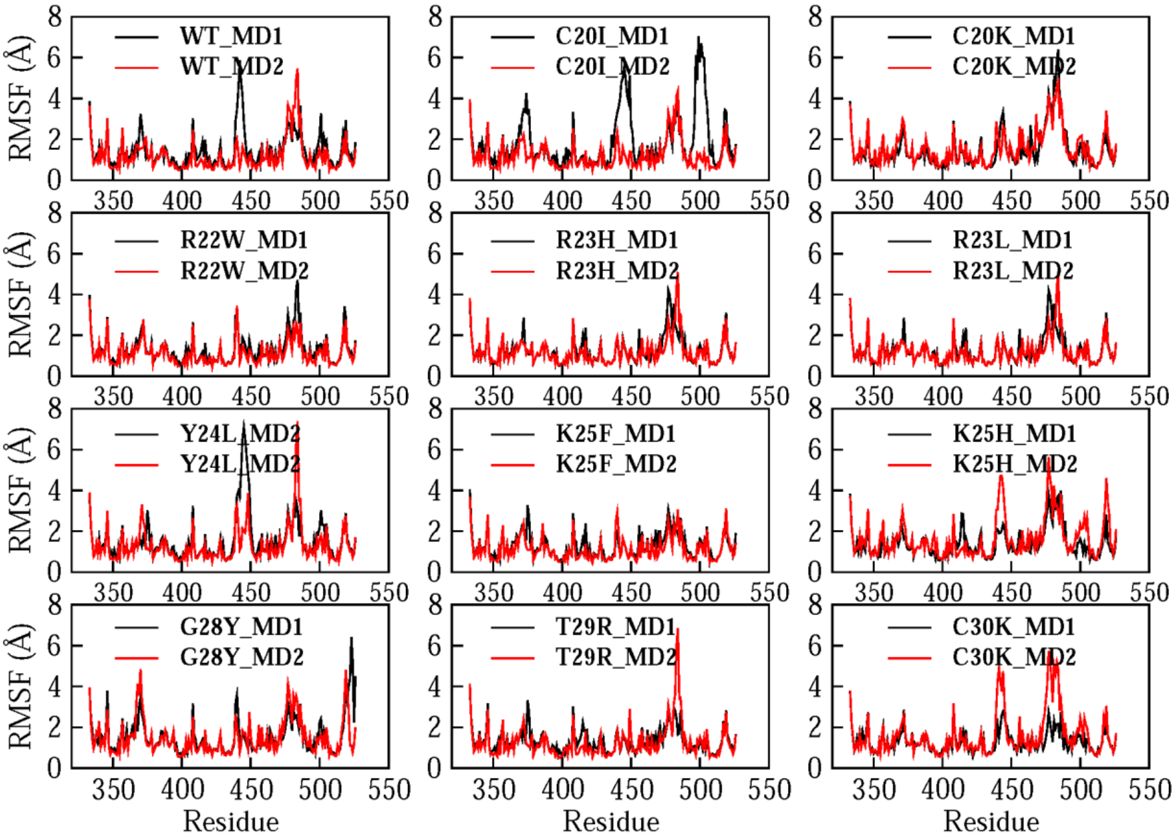

Figure S9: The residue-wise RMSF plot of RBD bound with hBD-2 wildtype and 11 point-mutants in both MD1 (in black) and MD2 (in red) simulations.

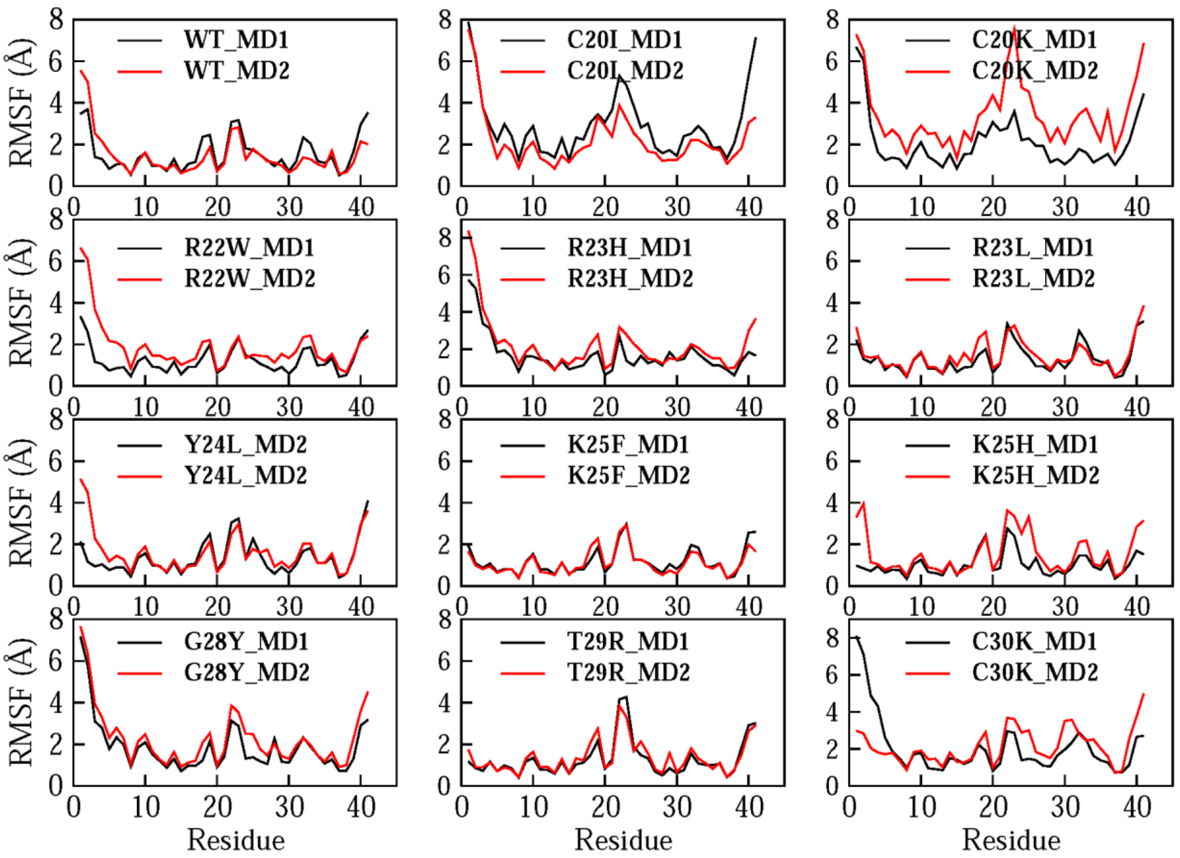

Figure S10: The residue-wise RMSF plot of hBD2 wildtype and 11 point-mutants bound with RBD in both MD1(in black) and MD2 (in red) simulations.
